# Nano Sensing and Energy Conversion Using Surface Plasmon Resonance (SPR)

**DOI:** 10.3390/ma8074332

**Published:** 2015-07-16

**Authors:** Iltai (Isaac) Kim, Kenneth David Kihm

**Affiliations:** 1 School of Engineering and Computing Science, Texas A&M University-Corpus Christi, 6300 Ocean Drive Unit 5797, Corpus Christi, TX 78412, USA; 2 Department of Mechanical, Aerospace, and Biomedical Engineering, University of Tennessee, 408 Dougherty Hall, Knoxville, TN 37996-2210, USA; E-Mail: kkihm@utk.edu

**Keywords:** surface plasmon resonance (SPR), nanophotonics, sensing, energy conversion

## Abstract

Nanophotonic technique has been attracting much attention in applications of nano-bio-chemical sensing and energy conversion of solar energy harvesting and enhanced energy transfer. One approach for nano-bio-chemical sensing is surface plasmon resonance (SPR) imaging, which can detect the material properties, such as density, ion concentration, temperature, and effective refractive index in high sensitivity, label-free, and real-time under ambient conditions. Recent study shows that SPR can successfully detect the concentration variation of nanofluids during evaporation-induced self-assembly process. Spoof surface plasmon resonance based on multilayer metallo-dielectric hyperbolic metamaterials demonstrate SPR dispersion control, which can be combined with SPR imaging, to characterize high refractive index materials because of its exotic optical properties. Furthermore, nano-biophotonics could enable innovative energy conversion such as the increase of absorption and emission efficiency and the perfect absorption. Localized SPR using metal nanoparticles shows highly enhanced absorption in solar energy harvesting. Three-dimensional hyperbolic metamaterial cavity nanostructure shows enhanced spontaneous emission. Recently ultrathin film perfect absorber is demonstrated with the film thickness as low as ~1/50th of the operating wavelength using epsilon-near-zero (ENZ) phenomena at the wavelength close to SPR. It is expected to provide a breakthrough in sensing and energy conversion applications using the exotic optical properties based on the nanophotonic technique.

## 1. Introduction

Recent sensing and energy conversion applications are reviewed using surface plasmon resonance and nanophotnic techniques. Nanophotonic techniques have been actively studied and applied in various fields from sensing, subwavelength imaging, to energy conversion in the last decade [1,2,3,4,5,6,7,8,9,10,11,12,13,14]. One popular approach in sensing application is to use surface plasmon resonance (SPR) technique [15]. SPR can be divided into two groups. One is planar SPR based on thin film [3,4,5,6,7] and the other is localized SPR based on nanoparticles [1,2]. Nanoparticle-based localized SPR has been developed in biosensing applications because of its simple schematic, while thin film based planar SPR shows higher sensitivity. There are various techniques developed in planar SPR; intensity change at a fixed incident angle, intensity change with angle, spectrogram, and interferogram measurements [14,16]. Recent review of planar SPR techniques is thoroughly summarized in Reference [17]. Intensity measurement at a fixed angle approach has several advantages compared with other methodologies. It can provide real-time and full-field imaging, which is beneficial to monitor near-surface transport or optical property changes such as concentration, temperature, salinity, and effective refractive index [3,4,5,6,7], which is not possible with existing techniques. This technique can be more powerful if its sensitivity can be increased in order to monitor the fine ion change in bio applications through grating structure [18] and high refractive index sheets [19]. Another idea is to combine the exotic optical properties of hyperbolic metamaterials (HMM) with its SPR resonance dispersion controllability to improve sensitivity [20].

Energy conversion is other main applications using nanophotonics. Nanaoparticle-based localized SPR plays a key role in hybrid nano-bio structures to enhance absorption efficiency of light-acceptor materials, such as photosystem I molecules, in energy harvesting by amplifying the surrounding electric field of metal nanoparticles when lights are illuminated [21]. This enhanced absorption efficiency can be applied to photoelectrochemical cells, photoelectronics, and hydrogen production [22,23,24]. Recently, hyperbolic metamaterial (HMM) has been gaining increasing attention as it shows enhanced energy transfer rate when dipole is close to its HMM surface experimentally and numerically [13,25,26,27,28]. Furthermore, ultra-thin films with deep subwavelength thickness have been shown to have perfect absorption using epsilon-near-zero (ENZ) phenomena at the wavelength close to SPR, which is appealing for device applications as surface patterning or texturing is not required and it needs lower material consumption [29].

## 2. Sensing Application using SPR Imaging

**Figure 1:**
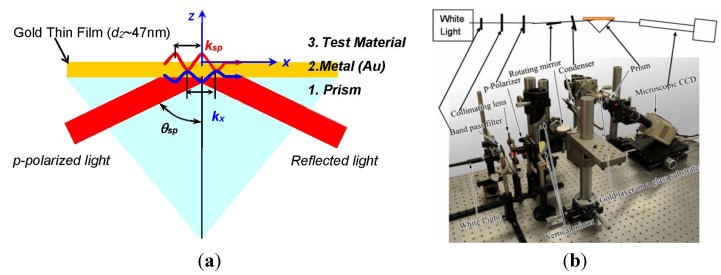
Surface plasmon resonance (SPR) system: (**a**) Kreschmann configuration of a three-layered SPR principle, where the glass prism, the metallic gold film and the test medium are labeled 1, 2, and 3, respectively, and (**b**) the experimental layout of the SPR imaging system using a *p*-polarized white light source [5]. Reprinted with permission from [6]. Copyright 2015 Elsevier Science Direct.

Surface plamson resonance technique has been actively employed for its high sensitivity in measuring refractive index variation with the order of 10^−^^5^ in refractive index unit [5,14]. Figure 1 shows the experimental illustration of SPR imaging system implemented in the laboratory of the University of Tennessee, which is recently implemented at Texas A&M University-Corpus Christi, following Kretschmann configuration composed of prism, Au-coated substrate, and test material. When a thin metal film is illuminated by a coherent *p*-polarized light at an incident angle exceeding the critical angle for total internal reflection, the evanescent wave vector (${\vec{k}}_{x}$) is formed at the incident (bottom) metal surface that successively triggers coherent fluctuations of free electrons at the surface of the metal film [5,15]. This coherent energy conversion of the photons into free electrons is called the surface plasmon (SP) phenomenon. The resonant excitation of SP in the laterally heterogeneous interface occurs when the condition of momentum matching is fulfilled, *i.e.*, ${\vec{k}}_{x}={\vec{k}}_{sp}$, where ${\vec{k}}_{x}$ is the evanescent wave vector along the surface. As a result of the SPR excitation and absorption of the incident light into the metal film, the reflected light intensity is darkened and is ideally nullified at the specified SP angle [5,15]. The parameters determining the SP angle include the incident light wavelength, the type and thickness of the metal film, the refractive index values of the metal layer and the prism, and the refractive index of the test medium. Thus, once the SP angle is set for the base material with the specified conditions showing the darkest background image, *i.e.*, the so-called SPR condition at the resonance of surface plasmon, any local changes of refractive index distribution in the test field will reduce the local SPR absorption and change the corresponding SPR reflectance. This is the key idea to non-intrusively detect transport and optical properties with fine spatial measurement resolution and high accuracy in real-time.

**Figure 2:**
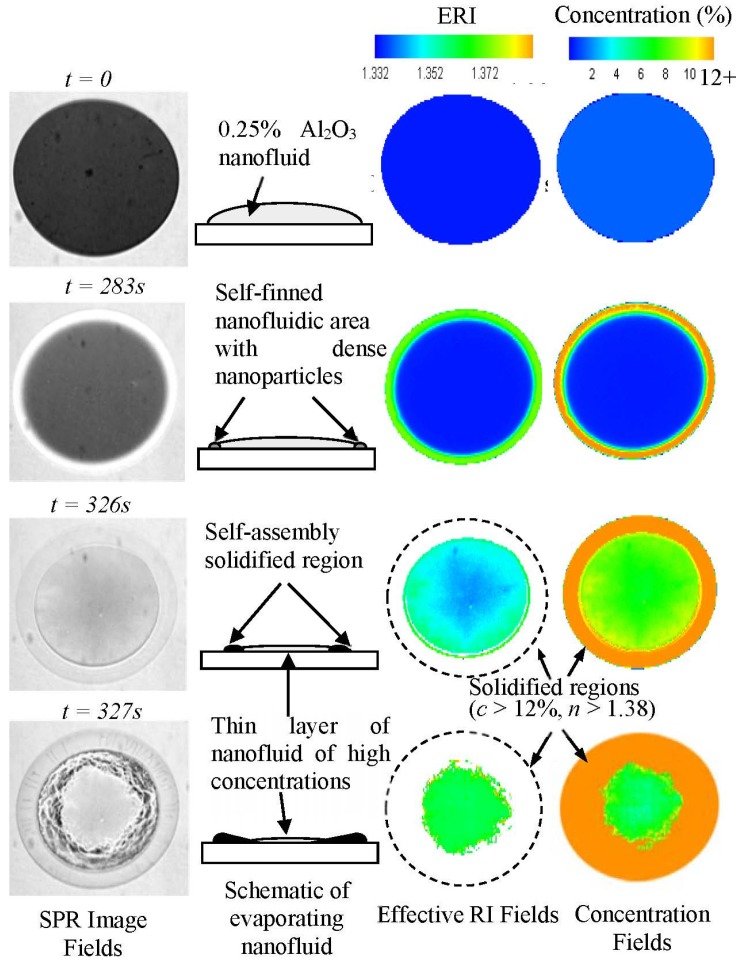
Full-field and real-time mapping of effective refractive index (ERI) and volume concentration distributions [4].

**Figure 3:**
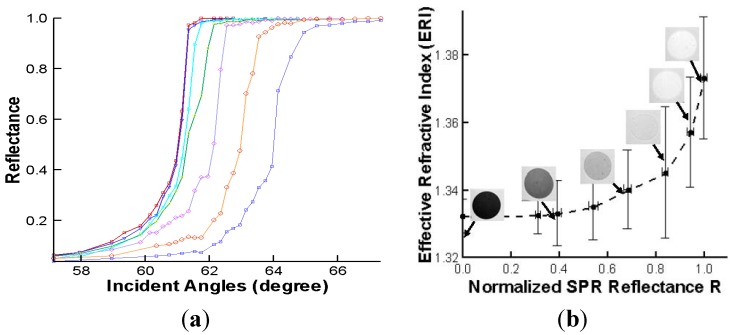
(**a**) Experimental determination of ERI of nanofluids using total internal reflection (TIR) and (**b**) correlation of TIR with normalized SPR reflectance *R* for varying concentrations [4].

Figure 2 shows *in situ* visualization of evaporation-induced self-assembly process at the low concentration of 0.25% Al_2_O_3_ nanofluids dispersed in deionized water using SPR imaging [4]. It presents *in situ* visualization of varying nanofluidic concentrations and the corresponding effective refractive index (ERI) of evaporating nanofluids. The dry-out pattern shows the traditional coffee-ring pattern due to self-pinning along the edge of droplets. The SPR imaging technique shows that it can detect the optical property variation of effective refractive index in a label-free and quantitative manner. The effective refractive index of nanofluids was successfully measured through total internal reflection (TIR) and verified using SPR reflectance imaging, as shown in Figure 3. Firstly, the effective refractive index (ERI) is determined from the TIR experiment and its measurement is in quite good agreement with the Rayleigh scattering theory. Then, experimental correlation for ERI is obtained between SPR reflectance and ERI to show that SPR imaging can successfully measure ERI of nanofluids. Unresolved issues in the optical characterization of nanofluids are the effective refractive index at high concentrations [4] and the change of optical property during the solidification of nanofluids [3]. The approach of higher refractive index prism, tunable SPR resonance wavelength, and enhanced sensitivity is expected to address these issues [18,19,20].

**Figure 4:**
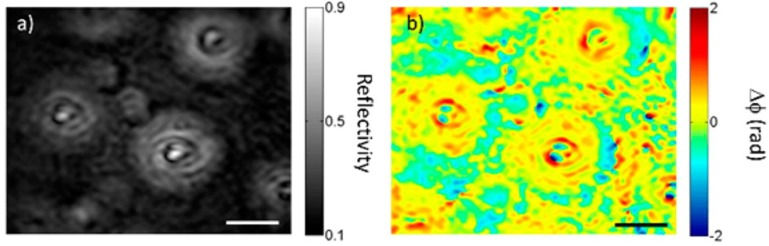
SPR holography shows SPR reflectance image (**a**) and phase contrast image (**b**) for the photoresist dome pattern. Scale bar is 10 μm. Reprinted with permission from [16]. Copyright 2015 American Chemical Society.

Very recently, SPR holography technique is demonstrated to record the SPR reflectance imaging and phase at the same time as in Figure 4, which is helpful in thin film material characterization. Figure 4 shows the comparison of SPR image and its corresponding phase for the dome pattern made of photoresist. The authors claim that both the SPR image (Figure 4a) and phase contrast image (Figure 4b) show a good contrast of photoresist residue between the two domes, while the phase contrast image emphasizes the imperfections at the top of domes. This method has the advantage of recording imaging and phase at the same time, but requires a complex optical set-up, while the technique of SPR imaging, introduced initially, is quite compact to implement and is more effective, especially in the case of samples requiring *in situ* detection.

**Figure 5:**
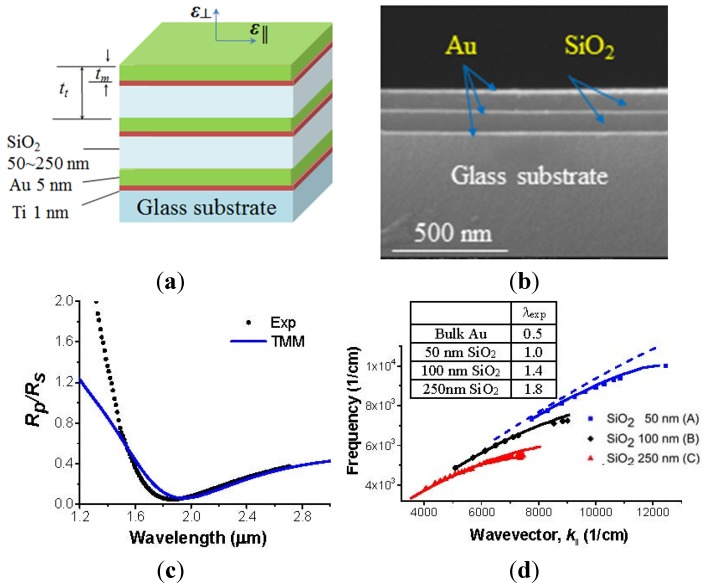
Schematic and SEM image of hyperbolic metamaterial (HMM) and the classic signatures for the identification of SPR mode of Hyperbolic metamaterial (2.5 pairs of Au/SiO_2_ with Au 5 nm and SiO_2_ 100 nm thickness) at incidence angle of 45 degree. (**a**) HMM made of alternating layers of Ti-Au (1 nm thick Ti and 5 nm thick Au), and SiO_2_ (50, 100 or 250 nm thickness); referred to sample A, B, and C, respectively. (**b**) Cross-sectional SEM image showing clear identification of 3 layers of Au (5 nm thick) and 2 layers of SiO_2_ films (100 nm thick). (**c**) The presence of a reflectivity minimum at the SPP resonance total internal reflection (TIR) configuration. The results obtained from the transfer matrix method (TMM) are a blue solid line. (**d**) Surface plasmon dispersion curves from three different metal filling factor samples (A, B, and C) of 0.118, 0.076, and 0.032 arising from a different SiO_2_ thickness: 50 nm in blue, 100 nm in black, and 250 nm in red, respectively. Experimental data from samples A, B, and C are indicated by symbols of squares (blue online), diamonds (black online), and triangles (red online). Dispersion calculation using TMM is solid. The free space light line is indicated by a light dotted line. The inset table summarizes the experimentally obtained shortest SPP wavelengths for the three samples [20].

Currently metallo-dielctric hyperbolic metamaterials (HMM) have attracted growing interest in a vast range of applications, including sub-wavelength imaging, spontaneous emission enhancement, and near-field energy transfer, because of their unique optical properties and simple fabrication. Spoof surface plasmon resonance, based on HMM, demonstrates effective SPR dispersion or wavelength control and exotic optical properties, which can be combined with SPR imaging for the selection of the SPR wavelength, depending on samples, providing higher sensitivity and enhanced spatial resolution. Figure 5a,b show a schematic and SEM image of hyperbolic metamaterials (HMM) comprised of smooth films (Au metal and SiO_2_ dielectric) with a very low metal filling factor [20]. HMM is fabricated by the e-beam evaporation technique. Figure 5c,d present the classic signatures for the identification of SPR mode of hyperbolic metamaterial (2.5 pairs of Au/SiO_2_ with Au 5 nm and SiO_2_ 100 nm thickness) at an incidence angle of 45 degree. The signatures and dispersion control of surface plasmon resonance from 1 to 1.8 μm is experimentally demonstrated using a periodic multilayer metallo-dielectric structure. The dispersion control of SPR, by simple variation of filling factor between metal and dielectric materials, provides extensive convenience to select the correct resonance wavelength of the test medium in nano-bio-chemical sensing applications. In addition, it is expected to suggest new sensing techniques using the exotic optical properties of HMM.

## 3. Energy Conversion Applications

**Figure 6:**
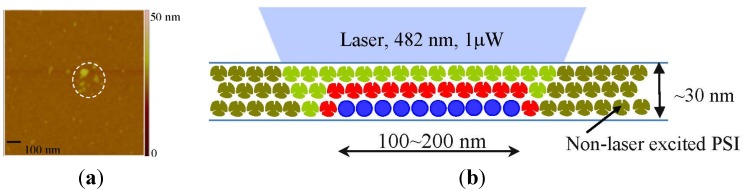
AFM image of large, aggregated Ag nanoparticles (**a**) and conceptual drawing of laser illumination of Ag nanoparticles coated by a PSI thin film (~30 nm thickness) (**b**). Reprinted with permission from [21]. Copyright 2015 American Chemical Society.

**Figure 7:**
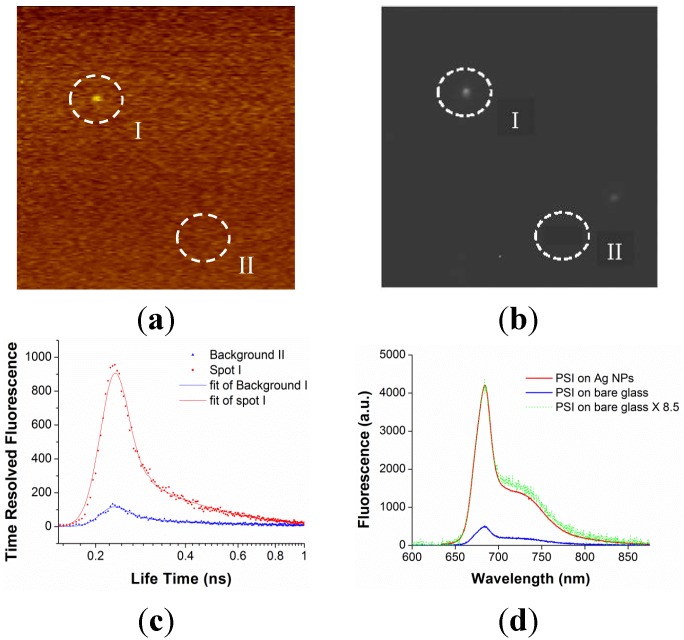
Correlation of PSI fluorescence and metal nanoparticle dark field scatting. (**a**) A confocal scanning microscope image of a 20 μm × 20 μm region of dilute nanoparticle (NP)-supported slide overlaid with a 30 nm thick PSI film. (**b**) A NP dark-field scattering image for the same region as (**a**). (**c**) A time-correlated, single photon correlated fluorescence amplitudes and lifetime decays measured using a 680 nm band pass filter for the same areas marked in (**a**) and (**b**). (**d**) An enhanced fluorescence through spectrograph for a PSI thin film on a single individual Ag spot and bare glass substrate excited at 482 nm with 100× objective and excitation laser power of 5 μW. Reprinted with permission from [21]. Copyright 2015 American Chemical Society.

Furthermore, nano-biophotonics could enable innovative energy conversion such as the increase of absorption and emission efficiencies and the perfect absorption. Localized SPR using metal nanoparticles shows highly enhanced absorption in solar energy harvesting. Figure 6 and Figure 7 show the result of absorption enhancement of photosystem I (PSI) molecules using Ag metal nanoparticles. PSI molecules have been attracting increasing attention because of its large voltage generation (~1 V), nanoscale resolution (~30 nm), stability, and low cost as a next generation materials for photoelectrochemical cells, photoelectronics, and hydrogen production. When light is illuminated to metal nanoparticles, metal nanoparticles generate strongly amplified electric field due to localized SPR and this strong electric field contributes to the absorption enhancement of its neighboring materials, such as PSI. Excited dynamics calculation shows that absorption enhancement is dominant compared with emission enhancement. The measured fluorescence is directly proportional to the absorption enhancement [21]. Figure 6a shows an AFM image of aggregated Ag nanoparticles coated on the glass substrate, and Figure 6b shows a conceptual drawing of laser illumination of Ag nanoparticles coated by a PSI thin film (~30 nm thickness). Figure 7 presents a correlation of PSI fluorescence and metal nanoparticle dark field scatting image for the same region in a sample, by sequentially applying confocal laser scanning, darkfield scattering imaging, time correlated single photon counting (TCSPC), and enhanced fluorescence measurement using a spectrometer. Region II shows a 10-fold enhanced fluorescence compared with region I, which does not show any enhanced fluorescence. Region II has aggregated Ag nanoparticles, as verified by Figure 7b, which provide strongly amplified electric signal to increase the absorption efficiency of PSI molecules coated on top of Ag nanoparticles. The significant increase of absorption (5- to 100-fold) can provide a substantial step toward optimizing nano-bio hybrid nanostructure in energy harvesting.

**Figure 8:**
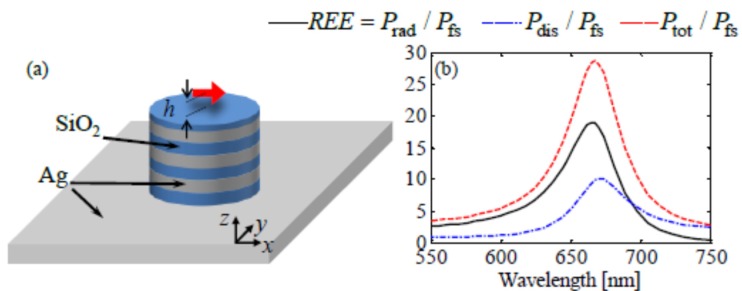
The illustration of the multilayer HMM resonator on top of Ag substrate (**a**) and enhancement of power emitted relative to the power of the same dipole radiated in free-space (**b**). Reprinted with permission from [25]. Copyright 2014, AIP Publishing LLC.

The unique optical property of hyperbolic metamaterials has been actively applied to enhance emission efficiency when HMM has three-dimensional structure. These three-dimensional HMM nanostructure can be fabricated with high production yield and uniformity, while the other approach of chemical synthesis to enhance emission efficiency is lacking in systematic and uniform production [21]. Recently, it has been shown that spontaneous emission can be enhanced dramatically by an emitter near a hyperbolic metamaterial substrate dramatically due to very large density of states [13,25,26,27,28]. Guclu *et al.* [25] numerically demonstrate that there is approximately a 100-fold enhancement of the free space radiative emission at 660 nm wavelength by hyperbolic metamaterials (HMM) resonator. HMM nano-antenna is designed to resonate at the wavelength of 660 nm, which is accessible using CdSe QDs with three pairs of silver (Ag) and silica glass (SiO_2_) layers (Figure 8a) with a radius of 54 nm and a height of 80 nm on top of opaque Ag coated substrate. Radiation power is calculated as in Figure 8b, to show that the total power is enhanced 30-fold due to the Purcell effect at the antenna resonance, peaking around 660 nm. Experimental verification efforts are under way at Sandia National Lab, where one of authors has worked. This result shows that a three-dimensional hyperbolic metamaterial (HMM) structure, based on nanophotonics, can be actively applied in energy conversion application.

**Figure 9:**
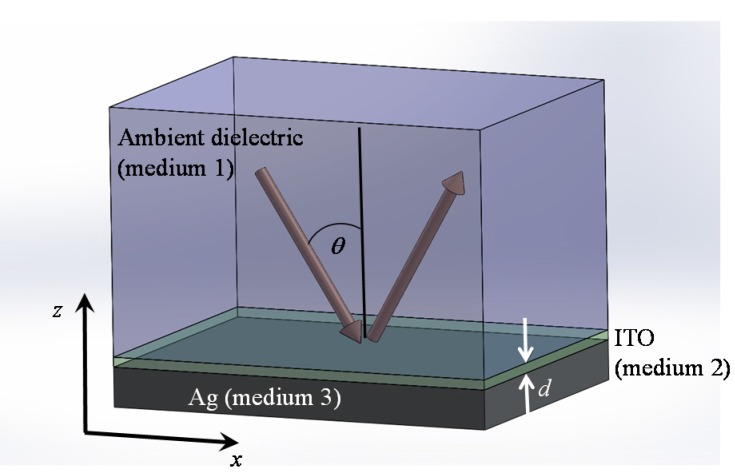
Schematic depiction of a three-medium structure comprising an ambient dielectric (medium 1), a subwavelength ITO layer of thickness *d* (medium 2) and a metallic substrate (medium 3). Reprinted with permission from [29].

**Figure 10:**
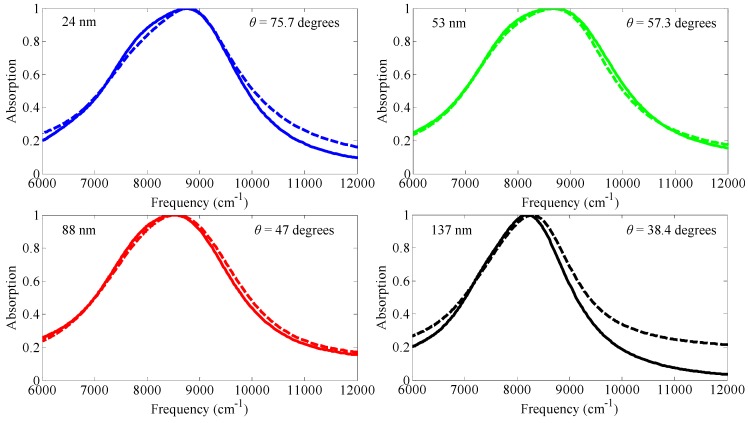
Comparison of measured perfect absorption profiles (solid) and calculated profiles using the three-medium Fresnel equation (dashed) for the four samples. The perfect absorption (PA) angle is explicitly indicated in each subplot. Reprinted with permission from [29].

The other energy conversion application is perfect absorption with low-loss and ultra-thin materials using the epsilon-near-zero (ENZ) phenomena near SPR wavelength region, as in Figure 9. This result can provide a path for the design of custom absorber materials without surface patterning, which is more preferable for practical device applications and further expand the list of possible material candidates for use in perfect absorption applications, to include conducting oxides, heavily doped semiconductors, and correlated electron materials such as vanadium dioxide. Figure 9 shows a schematic of perfect absorption with low permittivity materials (ITO film) whose thickness is as low as ~1/50th of the operating free-space wavelength, which is against common thought that a thicker film is needed for maximum absorption [29]. The ITO films were deposited on boroaluminosilicate glass substrates using a 90 wt % In_2_O_3_ /10 wt % SnO_2_ sputtering target at room temperature under a base vacuum pressure of 10^−7^ torr, followed by a 10-minute anneal in Ar gas at 700 °C. Four samples were prepared with differing ITO thicknesses of *d* = 24, 53, 88, and 137 nm. Subsequently, a 200 nm layer of Ag was deposited on the ITO films using electron beam evaporation. The absorption spectra obtained at the incidence angles for which the maximum absorption was obtained are shown in Figure 10, along with theoretical spectra computed using the three-medium Fresnel equation [30] (see Equation (1) in Section 3) with the measured ITO permittivity and the literature values for the permittivity of Ag [31]. A good agreement is observed between experimental and theoretical results. The maximum absorption values measured for the four samples are between 99.79%–99.98%.

## 4. Summary

Sensing application demonstrates that SPR imaging can detect the effective optical properties of nanofluids during evaporation-induced self-assembly process in label-free, real-time, and full-field manner. With the development of hyperbolic metamaterials (HMM), it is also shown that the one of the main parameters controlling SPR imaging, SPR wavelength, can be selectively chosen depending on the sample by simply adjusting the filling factor between the metal and dielectric films in HMM. The unique optical property of HMM is expected to provide a new sensing scheme with higher sensitivity. Furthermore, energy conversion applications, such as solar energy harvesting and perfect absorption, are presented to increase energy transfer rate with metal nanoparticles using localized SPR, three-dimensional hyperbolic metamaterial cavity, and ultra-thin film based on epsilon-near-zero (ENZ) phenomena.
